# Bridging the gap: heparan sulfate and Scube2 assemble Sonic hedgehog release complexes at the surface of producing cells

**DOI:** 10.1038/srep26435

**Published:** 2016-05-20

**Authors:** P. Jakobs, P. Schulz, C. Ortmann, S. Schürmann, S. Exner, R. Rebollido-Rios, R. Dreier, D. G. Seidler, K. Grobe

**Affiliations:** 1 Institute for Physiological Chemistry and Pathobiochemistry and Cells-in-Motion Cluster of Excellence (EXC1003-CiM), University of Münster, Waldeyerstr. 15, D-48149 Münster, Germany; 2 Center for Medical Biotechnology#, University of Duisburg-Essen, 45117 Essen, Germany; 3 Centre for Internal Medicine, Hannover Medical School I3, EB2/R3110, Carl-Neuberg-Str. 1, 30625 Hannover, Germany

## Abstract

Decision making in cellular ensembles requires the dynamic release of signaling molecules from the producing cells into the extracellular compartment. One important example of molecules that require regulated release in order to signal over several cell diameters is the Hedgehog (Hh) family, because all Hhs are synthesized as dual-lipidated proteins that firmly tether to the outer membrane leaflet of the cell that produces them. Factors for the release of the vertebrate Hh family member Sonic Hedgehog (Shh) include cell-surface sheddases that remove the lipidated terminal peptides, as well as the soluble glycoprotein Scube2 that cell-nonautonomously enhances this process. This raises the question of how soluble Scube2 is recruited to cell-bound Shh substrates to regulate their turnover. We hypothesized that heparan sulfate (HS) proteoglycans (HSPGs) on the producing cell surface may play this role. In this work, we confirm that HSPGs enrich Scube2 at the surface of Shh-producing cells and that Scube2-regulated proteolytic Shh processing and release depends on specific HS. This finding indicates that HSPGs act as cell-surface assembly and storage platforms for Shh substrates and for protein factors required for their release, making HSPGs critical decision makers for Scube2-dependent Shh signaling from the surface of producing cells.

The Sonic hedgehog (Shh) morphogen plays crucial roles in development^1^, but also contributes directly to the progression of various cancers^2^,^3^,^4^. The understanding of Shh function is therefore of great interest. Notably, production of active Shh protein begins with autocatalytic cleavage of a precursor molecule linked to the addition of a cholesteryl moiety to glycine 198 of the N-terminal Shh cleavage product^5^. This reaction is catalyzed by the C-terminal cholesterol transferase domain (ShhC). Next, a palmitoyl group is attached to the N-terminus of the N-terminal signaling domain (Fig. 1a). Hedgehog (Hh) N-acylation requires the expression of a separate gene product called Hh acyltransferase (Hhat)^6^,^7^,^8^,^9^,^10^. Hh palmitoylation is special in that the palmitate is attached via an amide bond to the α-amino group of the N-terminal cysteine, in contrast to O-acylation, which targets the serine hydroxyl side chain in Wnt proteins^11^, or S-acylation, which targets the thiol side chain in nearly all other palmitoylated proteins^10^,^12^. Hh palmitoylation during synthesis is critical for later signaling. Mutation of the N-terminal cysteine to serine or alanine (C > A/S) results in mutant forms that do not undergo palmitoylation^12^ and that show reduced patterning activity comparable to the respective acyltransferase-deficient mutants^7^,^13^,^14^,^15^,^16^,^17^. We refer to the dual-lipidated, fully active morphogen as Hh/Shh, whereas HhN/ShhN denotes the uncholesterylated N-terminal signaling domain that can be artificially generated by ShhC deletion.

As a consequence of their dual lipidation, Hhs tether to the surface of producing cells and bind to and multimerize on heparan sulfate (HS)-proteoglycans (HSPGs)^18^. Most cell types in vertebrates and invertebrates produce HSPGs, consisting of a core protein to which several HS chains are attached. HS biosynthesis (as well as the synthesis of heparin, a highly sulfated form of HS) occurs in the Golgi compartment. Enzymes called exostosins synthesize the (GlcA1,4GlcNAc1,4)_n_ carbohydrate backbone, which is subsequently modified by sulfotransferases and a GlcA-C5 epimerase. Notably, HS is required for the activity of lipid-modified fly Hh^19^,^20^,^21^. The glypicans [Gpcs, HSPGs attached to the cell membrane by a glycosylphosphatidylinositol (GPI) anchor] also regulate Hh signaling^22^,^23^ by the same poorly defined molecular mechanisms. Two recent reports indicated that one such mechanism may involve HS-regulated disassembly of Hh/Gpc complexes at the cell surface because *Drosophila* HS Sulfatase 1 (DSulf1)^24^ and vertebrate Sulf1^25^ stimulate Hh production at the source. Indeed, Gpcs can directly control spatiotemporal Hh release from producing cells in an HS-dependent manner *in vitro* and *in vivo*^26^, supporting the idea that HSPGs act as assembly and storage sites for Hh ligands, but can also recruit factors required for their regulated release^27^,^28^.

We wondered whether the soluble glycoprotein Scube2 [signal peptide, CUB domain, epidermal growth factor (EGF)-like protein 2] is one release factor that is attracted to the Shh source cluster in such a way. Scube2 is a member of the *you* class mutants in zebrafish^29^ and plays key cell-nonautonomous roles in Shh release and signaling *in vitro* and *in vivo*^30^,^31^,^32^,^33^,^34^. In contrast, Scube2Δ, a truncation mutant lacking the C-terminal cysteine-rich and CUB domains, is inactive^31^,^32^,^35^ (Fig. 1b). Two mechanisms have been suggested to explain this. One model suggests that Scube2 extracts and transports lipidated Shh by direct, CUB-domain-dependent interactions with its cholesterol moiety^31^. CUB domains, however, derive their name from the complement subcomponents C1r/C1s, sea urchin protein with EGF-like domains (UEGF), and bone morphogenetic factor 1 (BMP1) and contribute to protease activities in these proteins^36^. In agreement with this, another model suggests co-operative proteolytic Shh release by Scube2 and ADAM (a disintegrin and metalloprotease) family sheddases^35^. ADAM sheddases are soluble or membrane-bound proteases that solubilize the extracellular domains of various membrane proteins^37^. In the case of the Hhs, ADAM family members 10, 12, and 17 function as Shh sheddases^38^,^39^,^40^ regulated by HSPG expression^26^ and Scube2^35^ at the surface of Shh-expressing cells. To us, these findings indicate a key decision-making role of HSPGs in the regulated recruitment and assembly of sheddases and the sheddase activator Scube2.

In this work, by pointing out the key role of HSPGs in Scube2-facilitated Shh solubilization, we highlight a novel level of Shh signaling regulation by the hierarchical evolution of Shh source properties. First, we confirm that Scube2-enhanced morphogen release is unequivocally linked to the proteolytic processing of both lipidated Shh termini. We also show that isolated Scube2 domains impair this process in a dominant-negative way, suggesting that Scube2 acts as an adaptor to link sheddases with their HSPG-associated substrates. Indeed, by combining biochemistry, confocal heteroprotein imaging and genetics, we demonstrate that Scube2 recruitment and activity require specific HSPG expression at the surface of Shh source cells and that clustered basic amino acids located in the Scube2 spacer domain associate the molecule with heparin and HS *in vitro*. Consistent with this finding, heparin competition or HS degradation strongly impairs Scube2-dependent Shh release from the cell surface; moreover, the release of acidic lipidated proteins not associated with HS is Scube2 independent. To our knowledge, hierarchical Shh/Scube2/sheddase association at the surface of morphogen-producing cells represents the first example of a signal-releasing extracellular signalosome, as defined by a multiprotein complex of signaling elements whose association and proteolytic activities are regulated by an HSPG scaffold. This raises the exciting possibility that HSPG-dependent decision making ensures the specificity and speed of ectodomain release for the numerous other sheddase substrates expressed on one given cell.

## Results

### Full-length Scube2 enhances dual Shh processing

**Figure 1:**
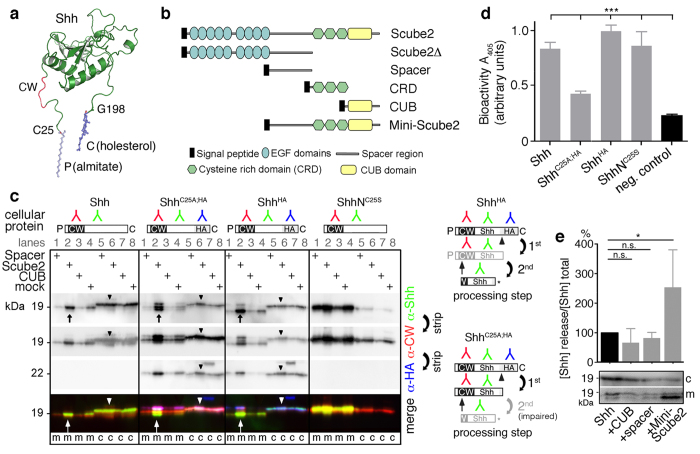
Scube2 increases proteolytic processing of Shh N-terminal lipidated peptides. (**a**) Modeled N-terminal palmitate (P) and C-terminal cholesterol (C) illustrate Shh membrane association by lipidated extended peptides (pdb: 1vhh). Lipidated amino acids and the CW motif are indicated. (**b**) Domain organization of Scube2 constructs used in this study. A FLAG epitope tag is present immediately after the signal peptide sequence for easy detection. (**c**) Scube2, but not the isolated spacer and CUB domains, increases lipidated peptide processing and Shh release. Palmitoylated Shh, Shh^HA^, and non-palmitoylated Shh^C25A;HA^ were expressed in Bosc23 cells in the presence or absence of Scube2 constructs. Proteins in the cellular (**c**) and corresponding soluble fractions (m) were analyzed by immunoblotting. To better demonstrate Shh processing during release, we inverted and colored the gray scale blots. Bright cellular signals in merged blots denote unprocessed proteins (arrowhead), yellow signals denote C-processed/N-unprocessed proteins, and green signals confirm the removal of N- and C-terminal peptides. Right: Schematic of Shh processing. Antibody binding sites, N-palmitate (P), C-cholesterol (C), and cleavage sites (arrow, arrowhead) are indicated. CW: Cardin-Weintraub motif, HA: hemagglutinin tag. Note that the N-terminal lipid impairs α–CW-antibody function. (**d**) C3H10T1/2 bioactivity assay of the same Scube2-released Shh processing products shown in C. Hh-regulated differentiation of pluripotent C3H10T1/2 cells into alkaline phosphatase-producing osteoblasts served as a readout. ***denotes statistical significance (p < 0.0001). (**e**) Quantification of Shh release by using ImageJ. Note that the isolated CUB and spacer domain reduce Shh release below baseline levels set to 100%, but that their physical linkage increases Shh release. Shh+CUB: 67% ± 21%, Shh+spacer: 82% ± 8%, Shh+Mini-Scube2: 254% ± 57%. *denotes statistical significance (p = 0.026). n.s.: not significant (p > 0.05). n = 5 for each data set.

Scube2 glycoproteins release dual-lipidated Shh (Fig. 1a) from transfected HEK293T cells^31^, HEK293S cells^32^, and Bosc23 cells^35^, a widely used HEK293 derivative. Like its homologs Scube1 and Scube3, Scube2 consists of a signal peptide for secretion and nine EGF domains linked by a spacer domain to a cysteine-rich domain and the C-terminal CUB domain (Fig. 1b). “Mini-Scube2,” which lacks all EGF domains, is still functional^32^. In contrast, Scube2Δ, which lacks the cysteine-rich and CUB domains, is inactive^30^,^31^,^32^,^35^,^41^,^42^. We compared Scube2-enhanced Shh release with the activities of the isolated spacer and CUB domains by SDS-PAGE and immunoblotting. To prove that Scube2 activity and shedding are unambiguously linked^35^, we C-terminally tagged Shh^HA^ and unlipidated Shh^C25A;HA^ with hemagglutinin (HA), resulting in the extended C-terminal membrane anchor N^190^SVAAKSG-*YPYDVPDYA*-G^198^ (G^198^ represents the cholesterol-modified glycine; underlined italicized letters represent the tag)^35^. We used bicistronic mRNA constructs for the coupled expression of all Shh constructs and Hhat in the same cells. α-CW antibodies raised against the CW peptide K^33^ RRHPKK^39^ located adjacent to the palmitoylated cysteine were used to detect N-terminal processing^43^. Polyclonal α-ShhN antibodies detected full-length and truncated proteins on the same (stripped) blot (Fig. 1c). To better demonstrate Shh processing during release, we inverted and colored the gray scale blots (green: α-Shh signal, red: α-CW signal, blue: α-HA signal). Bright cellular signals in merged blots thus denote unprocessed proteins, yellow signals denote C-processed/N-unprocessed soluble proteins, and green signals confirm the removal of N- and C-terminal peptides.

As shown in Fig. 1c, Scube2 increased the release of all lipidated Shh forms [compare lanes 2 (lipidated proteins + Scube) with lanes 4 (no Scube)], thereby converting cellular Shh and Shh^HA^ into truncated soluble morphogens. This is indicated by an electrophoretic size shift and lack of most α-CW and all α-HA antibody reactivity [compare the cellular (c) material in lanes 6 (arrowheads) with corresponding media in lanes 2 (arrow)]. On average, Shh solubilization increased about 16-fold (+1635% ± 200%, n = 12, p < 0.0001) in the presence of Scube 2 compared with Shh amounts released in its absence (set to 100%). Cell-surface-associated Shh^C25A;HA^ was C-terminally processed (yellow band, compare with Shh^HA^) but also dually processed (green band), demonstrating that cleavage of the non-palmitoylated N-terminus was impaired but not abolished^43^. Consistent with this, reduced but detectable Shh^C25A;HA^ bioactivity was observed in the Shh-responsive cell line C3H10T1/2^44^ [Fig. 1d: Shh: 0.84 ± 0.06 arbitrary units (au) (n = 12); Shh^C25A;HA^: 0.43 ± 0.02 au (n = 8); Shh^HA^: 1 ± 0.06 au (n = 12); ShhN^C25S^: 0.87 ± 0.13 au (n = 8); Mock: 0.24 ± 0.015 au (n = 12)]. The observed Hh-dependent differentiation of this cell line into alkaline phosphatase-producing osteoblasts demonstrates that N-terminal processing, and not N-palmitate per se, determines Shh biofunction (see also Supplementary Fig. S1). As expected, control ShhN^C25S^ lacking both lipids was secreted in a Scube2-independent, unprocessed form. ShhN^C25S^ bioactivity (Fig. 1d) is explained by its monomeric nature^40^ and high expression levels.

Notably, the isolated Scube2 spacer and CUB domains decreased Shh processing and release below the background level [Fig. 1c, compare lanes 4 (mock) with lanes 1 (spacer) and lanes 3 (CUB); and Fig. 1e]^32^, consistent with a previous observation^45^. This finding is in complete contrast to increased Shh processing and release by Mini-Scube2, which physically links both domains (Fig. 1e). This indicates that Scube2 is an adaptor that connects Shh sheddases with their substrates and that isolated domains compete with this activity by blocking the binding sites of the Shh substrate, sheddases, or scaffolding proteins at the cell surface^26^.

### Scube2 interacts with physiologically relevant HS

**Figure 2:**
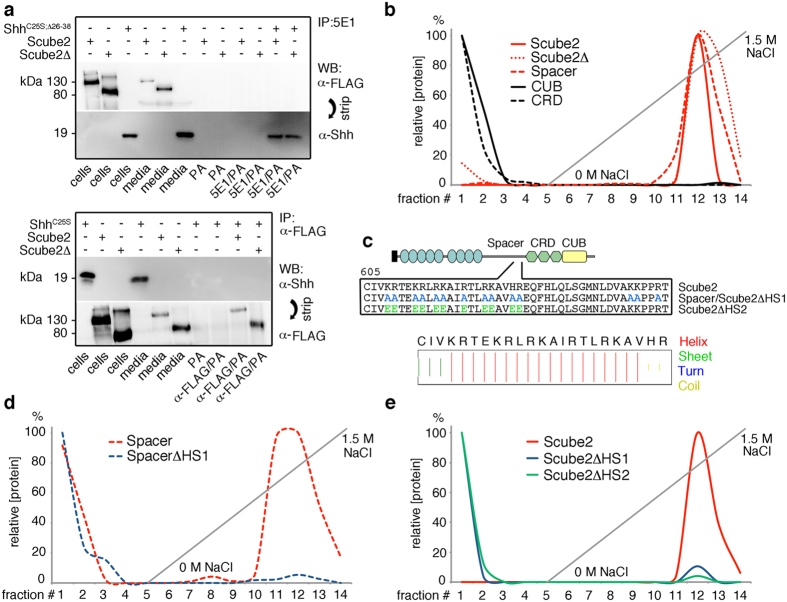
The Scube2 spacer domain binds to physiologically relevant HS. (**a**) Top: 5E1 immunoprecipitates Bosc23-expressed N-truncated Shh but does not co-immunoprecipitate Scube2 or Scube2Δ. The 130 kDa and 80 kDa signals from cells or trichloroacetic acid-precipitated media denote Scube2 and Scube2Δ, respectively. PA denotes Protein-A agarose beads; 5E1/PA denotes Shh-specific 5E1 antibodies coupled to PA beads; IP: immunoprecipitation; WB: Western blot. One representative result of three independent experiments is shown. Bottom: α-FLAG immunoprecipitates Scube2 and Scube2Δ, but does not co-immunoprecipitate full-length Shh^C25S^. One representative result of three independent experiments is shown. (**b**) Scube2 and Scube2 variants were applied to columns coupled with mouse embryonic HS. Whereas the CUB and CRD domains were found in the flow through and wash (fractions 1–4), Scube2, Scube2Δ, and the spacer domain tightly bound to the column, suggesting strong HS interactions. (**c**) HS-binding candidate amino acids (top) located in a helical spacer peptide (bottom) were replaced with alanines (ΔHS1) or glutamic acids (ΔHS2). This completely abolished HS binding of spacerΔHS1 (**d**) and full-length Scube2ΔHS1 and Scube2ΔHS2 constructs (**e**).

We first analyzed possible Scube2 binding to the Shh substrate. Shh-specific 5E1 antibodies were coupled to Protein A beads (5E1/PA) and incubated with soluble Scube2 or Scube2Δ in the presence or absence of N-terminally truncated Shh (Shh N-truncation facilitates 5E1 binding^40^). As controls, Scube2 or Scube2Δ in media were trichloroacetic acid (TCA) precipitated to confirm their solubilization, and both proteins were incubated with PA beads or 5E1/PA in the absence of Shh to rule out any nonspecific interactions. As shown in Fig. 2a (top), 5E1/PA beads immunoprecipitated Shh, but wild-type or mutant Scube2 was not co-immunoprecipitated^31^. The reciprocal experiment, using Scube2-specific α-FLAG antibodies coupled to PA beads (α-FLAG/PA), likewise failed to reveal Shh co-immunoprecipitation, demonstrating the absence of any Shh/Scube2 complexes in solution (Fig. 2a, bottom). We thus conclude that Scube2 recruitment to Shh release sites may be mediated by another constituent of the Hh-containing cell-surface cluster.

**Figure 3:**
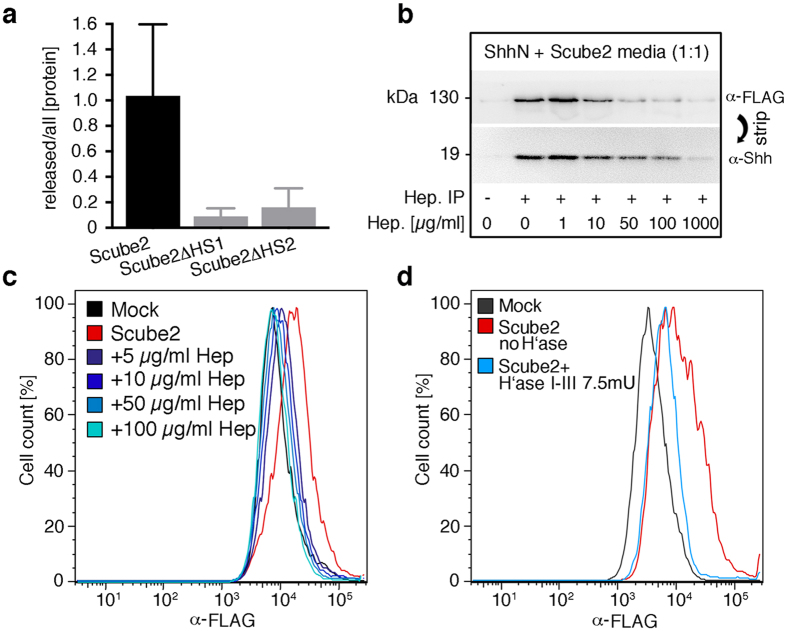
Soluble heparin and removal of cell-surface HS reduce Scube2 binding to Bosc23 cells. (**a**) Secretion of Scube2ΔHS1 and of Scube2ΔHS2 is reduced when compared with wild-type Scube2, consistent with spacer domain-dependent expression and release of the glycoprotein^70^. (**b**) Soluble heparin in the medium impairs soluble ShhN and Scube2 interactions with immobilized heparin, suggesting that HS can co-localize Scube2 and its ligand. Competing heparin amounts ranging from 0 μg/ml to 1000 μg/ml were used. (**c**) FACS of Scube2-expressing Bosc23 cells reveals that Scube2 associates with the cell surface when compared with a mock-transfected control. Heparin (5–100 μg/ml) specifically interfered with this interaction. A total of 50,000 cells were counted for each data set (see also Supplementary Fig. S4). (**d**) Cell-surface HS degradation by 7.5 mU heparinases I to III (2.5 mU each) impairs Scube2 binding to the cell surface. A total of 50,000 cells were analyzed (see also Supplementary Fig. S4).

On the basis of previously described Hh co-localization with HSPGs, we hypothesized that HS may play this role^18^,^26^. To test this idea, we isolated HS from mouse embryos and coupled the material to HiTrap columns for subsequent fast protein liquid chromatography (FPLC) (Fig. 2b and Supplementary Fig. S2). Expressed Scube2 and different Scube2 deletion variants (a kind gift of Ruey-Bing Yang, Academica Sinica, Taiwan) were loaded onto the HS-coupled column, and proteins were eluted by increasing salt concentrations. We observed quantitative Scube2 and Scube2Δ binding and protein elution at 1.1 M NaCl, indicating strong HS interactions. In contrast, the isolated CUB and cysteine rich domain (CRD) did not bind HS (Fig. 2b), but the isolated spacer bound HS. Sequence analysis identified a helical arrangement (Fig. 2c; bottom) of clustered basic amino acids (Fig. 2c; top) at the C-terminal portion of the spacer (Supplementary Fig. S3), representing a potential HS binding site. This was confirmed by site-directed mutagenesis (Fig. 2c). As expected, the exchange of all 14 basic amino acids for neutral alanines abolished all HS interactions of the resulting spacerΔHS1 construct (Fig. 2d) and of full-length Scube2ΔHS1 (Fig. 2e). The exchange of 11 basic amino acids for acidic glutamates (Scube2ΔHS2; Fig. 2c) had a similar effect (Fig. 2e). From these findings, we draw the conclusion that a 23-amino acid motif located in the Scube2 spacer domain is sufficient to bind Scube2 to HS, explaining its critical role for Scube2 membrane association^42^. Unfortunately, the secretion of mutant Scube2 variants was severely and variably impaired (Fig. 3a): Scube2ΔHS1 was released at only 10% ± 3% and Scube2ΔHS2 at only 15% ± 7% of wild-type control levels. This impairment prevented us from further characterizing them *in vivo* in the zebrafish.

### Binding of Scube2 to the cell surface is HS-sulfation dependent

We resorted to competition assays as an alternative approach to determine the importance of Scube2 binding to cellular HS. First, soluble Shh and Scube2 was pulled down by using immobilized heparin and analyzed by SDS-PAGE/immunoblotting. Specificity of the interaction was controlled by added soluble heparin. As shown in Fig. 3b, 1 μg of soluble heparin did not significantly impair protein co-localization on immobilized heparin. In contrast, 10 μg/ml soluble heparin reduced Scube2 binding to 29% and Shh protein binding to 71% of control levels, and 1 mg/ml soluble heparin almost completely blocked protein interactions with the immobilized form (2.4% of Scube2 and 6.2% of Shh levels if compared to those obtained in the absence of soluble heparin). This provides a proof-of-principle for HS-mediated Scube2/Shh co-localization.

**Figure 4:**
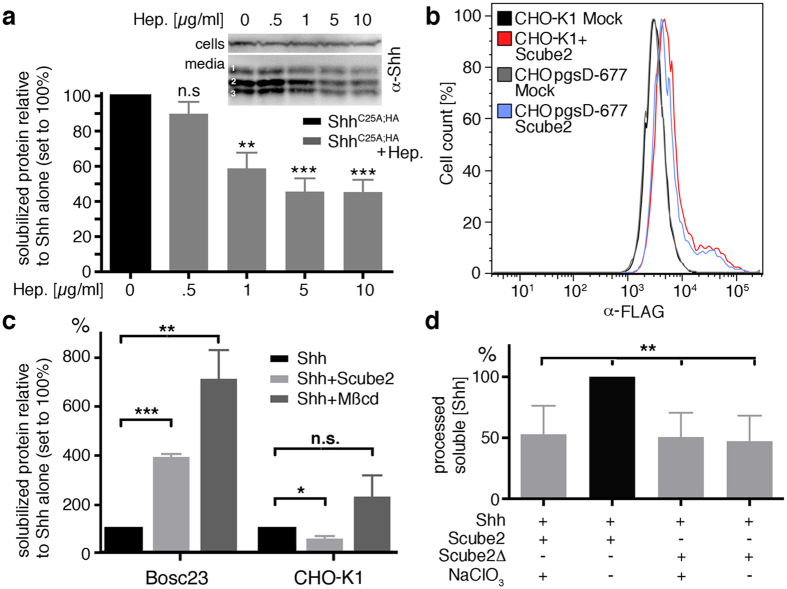
Scube2 HS binding and function depends on the expressing cell type. (**a**) Soluble heparin dose dependently suppresses Scube2-regulated N- and C-terminal Shh^C25A,HA^ processing from Bosc23 cells. A concentration of 1 μg/ml heparin was sufficient to largely inhibit C-terminal (band 2, inset) and dual processing (band 3, inset) (refer to the Shh^C25A,HA^ banding pattern and the underlying processing steps outlined in Fig. 1c). Band 1 denotes cellular material not removed by ultracentrifugation. ***p < 0.0001, **p < 0.005, n.s.: nonsignificant. n = 8–9. (**b**) Scube2 does not associate with the cell surface of CHO-K1 cells and HS-deficient CHO pgsD-677 cells. This finding suggests that specific cell-surface HS is required for Scube2 binding. (**c**) Scube2 strongly enhances Shh release from the surface of expressing Bosc23 cells, but not from CHO-K1 cells. Yet, Mβcd-forced shedding confirmed expression of Shh ligand and sheddases at the surface of both cell lines. ***p < 0.0001, **p = 0.0065, *p = 0.0158, n.s.: nonsignificant. n = 3. (**d**) Sodium chlorate decreases cell-surface HS sulfation and abolishes Scube2-dependent Shh^C25A,HA^ release. Shh release in the presence of Scube2 and sodium chlorate is comparable to Shh release in the presence or absence of Scube2Δ. **Denotes significance, p < 0.007, n = 4.

Scube2 is a secreted yet surface-associated glycoprotein^42^. To test whether this association depends on HSPGs, we stained Scube2 transfected Bosc23 cells under non-permeabilizing conditions with α-FLAG antibodies directed against Scube2 and quantified the binding by fluorescence activated cell sorting (FACS). FACS confirmed Scube2 association with the Bosc23 cell surface (Fig. 3c,d; Supplementary Fig. S4). To prove the specificity of the interaction, we preincubated the cells with heparin. We observed significantly decreased levels of Scube2 at the cell surface as a consequence of preincubation with 5–50 μg/ml heparin, and we completely abolished Scube2 cell-surface binding upon preincubation with 100 μg/ml heparin, which we explain as the competition of Scube2/HS interactions by soluble heparin. Consistent with this, HS digestion by heparinases I to III also decreased Scube2 amounts at the cell surface (Fig. 3d). We thus suggest that HSPGs can recruit soluble Scube2 to the cell surface. This may be essential for Scube2 biofunction. To test the role of HS binding for Scube2-regulated protein release, we added increasing amounts of soluble heparin to the media of Scube2- and Shh^C25A;HA^-expressing cells and quantified solubilized morphogen (Fig. 4a). Whereas 0.5 μg/ml heparin did not significantly affect Shh^C25A;HA^ solubilization (90% ± 6.6%, n = 8, p < 0.21) compared with protein amounts released in the absence of heparin (set to 100%), 1 μg/ml heparin decreased this solubilization by more than a third (59% ± 8%, n = 8, p < 0.0018) and heparin amounts exceeding 5 μg/ml decreased solubilization by more than half (5 μg/ml: 46% ± 8%, n = 8, p < 0.0001; 10 μg/ml: 45% ± 7%, n = 9, p < 0.0001). Soluble heparin thus blocked Scube2-dependent Shh processing from the cell surface. This finding suggests that HS-mediated Scube2 co-localization with its substrate is a prerequisite for Shh release and signaling from the producing cell surface.

### Scube2 binding and activity depend on the HS-expressing target cell type

To further prove that HS binds Scube2 to the cell surface, we expressed Scube2 in CHO pgsD-677 cells, a CHO-K1 line deficient in the synthesis of HS, but not the related glycosaminoglycan chondroitin sulfate (CS)^46^. Like HS, CS is a sulfated acidic polysaccharide, but because of structural differences, many proteins prefer to interact with HS. FACS of mock-transfected and Scube2-expressing CHO pgsD-677 cells revealed that only very limited amounts of Scube2 bound to the cell surface (Fig. 4b). Notably, HS-expressing CHO-K1 showed a comparable profile (Fig. 4b), despite abundant Scube2 secretion into the media (Supplementary Fig. S5). This observation suggests an important role of the fine structure of HS or the overall degree of HS sulfation in Scube2 binding. In this regard, we noticed that most data on Scube2 functions were obtained from HEK293 cells or their derivatives, such as Bosc23^31^,^32^,^35^,^42^,^45^, supporting the possibility that Scube2-regulated Shh release specifically depends on the expressing cell type. If correct, this finding would predict that Shh release from CHO-K1 cells is irresponsive to Scube2. We tested this idea by directly comparing Scube2-dependent Shh protein release from Bosc23 cells and CHO K1 cells in the same experiment. As shown in Fig. 4c, whereas Scube2 increased Shh release from the Bosc23 cell surface by about 4-fold in this assay (+390% ± 12%, proteins were released over night), Shh release from CHO-K1 cells was decreased (−56% ± 11%). Scube2 expression was confirmed on the same (stripped) blots, ruling out the possibility of expression or secretion artifacts (Supplementary Fig. S5). Finally, unimpaired expression of Shh and Shh sheddases at the CHO cell surface was controlled by 600 μg/ml methyl-β-cyclodextrin (Mβcd) added to the media (Fig. 4c). Mβcd nonspecifically activates cell-surface sheddases as a means to detect previously unreleased membrane-associated proteins^47^. Indeed, Mβcd solubilized previously unreleased Shh from the surface of Bosc23 cells (+710% ± 120% processed Shh) and from CHO-K1 cells (+229% ± 12%) (see also Supplementary Fig. S5). Therefore, the most likely explanation for Scube2 insensitivity in CHO-K1 cells is that HS-dependent Scube2 association with HSPG-linked Shh cell-surface clusters is impaired. This, in turn, interferes with the recruitment or activation of Shh sheddases at the sites of Shh storage.

To further test this scenario, we changed HS-dependent Scube2 recruitment and activity regulation by the chemical modification of Bosc23 cell-surface HS with sodium chlorate (NaClO_3_). NaClO_3_ inhibits the biosynthesis of the universal sulfate donor 3′-phosphoadenosine-5′-phosphosulfate, thereby reducing overall HS sulfation. This strongly impaired Scube2-dependent Shh release, but did not change Shh/Scube2Δ baseline release, indicating that NaClO_3_ was not toxic to cells (Fig. 4d). NaClO_3_ almost halved Shh release (53% ± 12%, Scube2-regulated release = 100%), a value close to the baseline of 48% ± 10% (Shh+Scube2Δ) and 51% ± 10% (Shh+Scube2Δ+ NaClO_3_), p = 0.007, n = 4. Thus, the overall degree of Bosc23 HS sulfation or the fine structure of Bosc23-produced HS regulates Shh-specific Scube2 recruitment and function^26^. We thus propose that Scube2 bridges the gap between HSPG-associated Shh substrates and their soluble or cell-surface-associated sheddases in an HS-specific manner.

**Figure 5:**
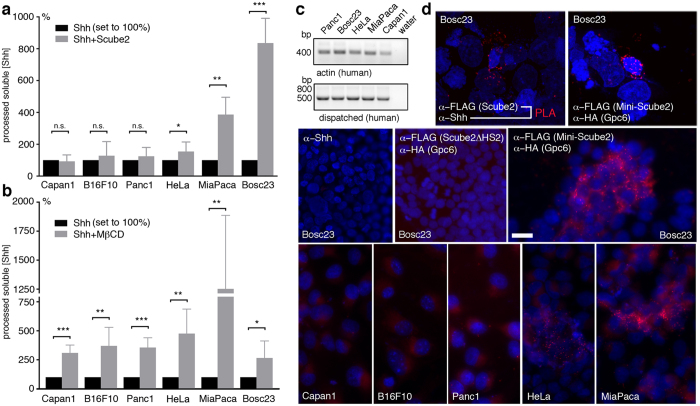
Correlated Scube2 function and HS binding at the cell surface. (**a**) Scube2 strongly enhances Shh release from Bosc23 cells, but not from Capan1, B16F10 and Panc1 cells. HeLa and MiaPaca2 cells show limited Scube2 sensitivity. ***p < 0.0001, **p < 0.005, *p < 0.02, n.s.: not significant. (**b**) Mβcd-forced shedding confirmed expression of Shh ligand and sheddases in all cell lines. ***p < 0.0001, **p < 0.005, *p < 0.02, n = 3–9 per line. (**c**) All human cells express comparable levels of dispatched, as determined by semiquantitative RT-PCR. Actin served as a loading control. (**d**) Detected Scube2/Shh interactions and Scube2/Gpc6 HSPG interactions at the cell surface by *in situ* proximity ligation assay (PLA). The top images show a maximum intensity projection of the raw image based on 20 Z-planes. All other images are fluorescent micrographs. Individual PLA signals as a consequence of Scube2/Shh interactions and Scube2/Gpc6 HSPG interactions are shown as red dots and nuclei are shown in blue. α-Shh alone and α-FLAG (Scube2ΔHS2)/α-HA (Gpc6) denote negative controls. Only occasional non-clustered PLA signals were observed at the surface of some Capan1, B10F10 and Panc1 cells; HeLa and MiaPaca2 cells, in contrast, showed numerous interactions. Size bar: 10 μm.

**Figure 6:**
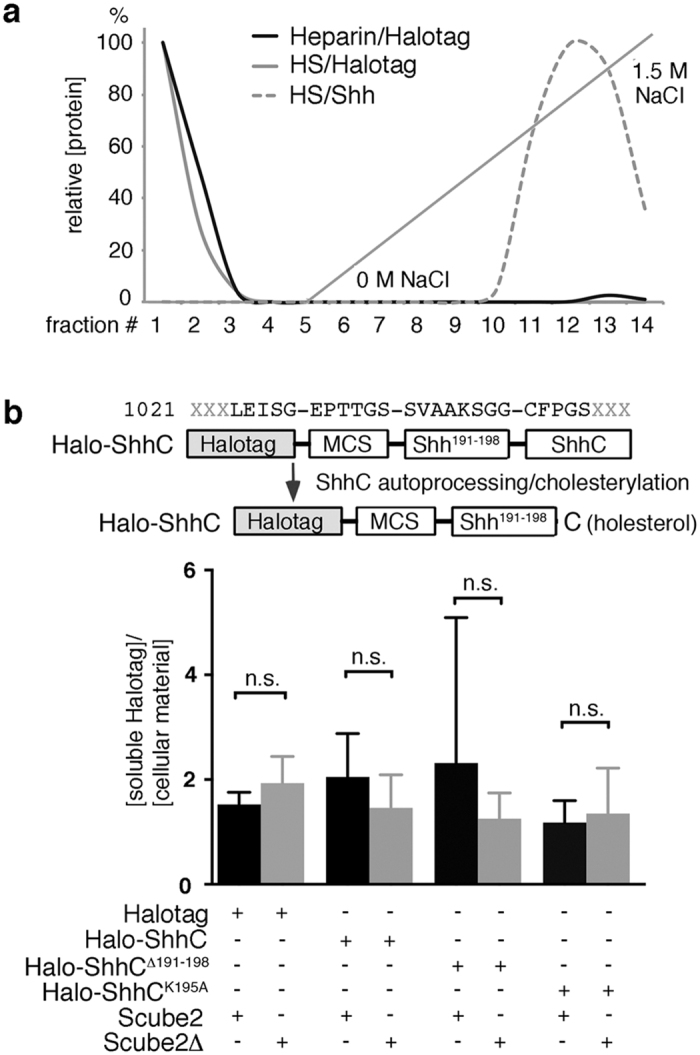
Scube2/HS association is required for Shh release. (**a**) Affinity chromatography reveals binding of positively charged Shh but not of negatively charged Halotag to heparin and HS. (**b**) Top: In addition to the soluble Halotag used above, various fusion constructs carrying C-terminal amino acids of the ShhN signaling domain and the C-terminal cholesteryltransferase domain^31^ were expressed. Autocleavage of this domain in the secretory pathway results in C-terminally cholesterylated Halotag proteins that tether to the plasma membrane of the cell. Bottom: The release of soluble Halotag and its cholesterylated variants is not affected by Scube2. Ratio of soluble/cell bound Halotag+Scube2: 1.53 ± 0.13, Halotag+Scube2Δ: 1.93 ± 0.29, C-terminally cholesterylated Halo-ShhC+Scube2: 2.05 ± 0.48, Halo-ShhC+Scube2Δ: 1.46 ± 0.36, Halo-ShhC^Δ190–197^+Scube2: 2.32 ± 1.60, Halo-ShhC^Δ190–197^+Scube2Δ: 1.25 ± 0.28, Halo-ShhC^K195G^+Scube2: 1.18 ± 0.24, Halo-ShhC^K195G^+Scube2Δ: 1.35 ± 0.50. n.s. = not significant. n = 3 for each data set.

To support this idea, we compared Scube2-regulated release of recombinant Shh from Capan1, B16F10, Panc1, HeLa and MiaPaca2 cancer cell lines by using the strategy outlined earlier (Fig. 1). HS composition differs between B16F1, B16Bl6, PC3 and HeLa cells^26^. Scube2 enhanced Shh release from Bosc23-positive control cells by about 8-fold (+835% ± 63%, n = 6, p = 0.0001) and from HeLa and MiaPaca2 cells by about 1.5-fold and 4-fold, respectively (HeLa: +155% ± 20%, n = 9, p = 0.014; MiaPaca2: +387% ± 54%, n = 4, p = 0.0019) (Fig. 5a). In contrast, Scube2 did not affect Shh release from Capan1, B16F10 and Panc1 cells (Capan1: 93% ± 23%, n = 3; B16F10: 128% ± 36%, n = 6; Panc1: 124% ± 28%, n = 4, p > 0.5 in all cases). Comparable Scube2 expression was confirmed on the same (stripped) blots, and the expression of Shh sheddases was confirmed by their nonspecific Mβcd stimulation. This increased Shh release from Capan1 cells by 3-fold, from B16F10 cells and Panc1 cells by about 3.7-fold, from HeLa cells by 5-fold and from MiaPaca2 cells by 12-fold (Fig. 6b). Consistent with these results, all human cell lines expressed mRNA for dispatched, an essential protein for the release of cholesterol-modified Hh^6^ (Fig. 5c).

Next, we used light microscopy to analyze HS-dependent Scube2 recruitment to these cell lines. However, diffraction-limited confocal microscopy has insufficient resolution to confidently demonstrate Scube2/Shh and Scube2/HSPG interactions at the cell surface. We thus resorted to an experimental design in which transfected cells were grown on coverslips, fixed, and probed with primary antibodies directed towards Scube2 and HSPGs or Scube2 and Shh. This was followed by incubation with two sets of secondary antibodies conjugated with specific oligonucleotides. Subsequent oligonucleotide ligation by a bridging probe in a proximity-dependent manner (40 nm being the upper limit of the interactions) was followed by rolling-circle amplification to visualize heteroprotein interactions as fluorescent spots. The specificity of the reactions was confirmed by using only one primary antibody in conjunction with both secondary proximity ligation assay (PLA) probes.

As shown in Fig. 5d and Supplementary movies M1, 2, 3, 4, individual signals obtained from confocal scans confirmed direct Shh and Scube2 interactions (158 cells showing >10 PLA signals at their surface were detected in a 1 mm^2^ area). This interaction was specific, because using only the α-Shh antibody in conjunction with both secondary probes (α-Shh) or only the α-FLAG antibody (not shown) failed to produce any PLA signals. We also observed increased direct interactions between glypican 6 (Gpc6) HSPGs and Mini-Scube2 at the surface of Bosc23 cells. Differences in Scube2 binding to Shh or HSPGs may indicate only transient Scube2/Shh interactions during morphogen release, yet more persistent HS binding. Consistent with this, Gpc6/Scube2ΔHS2 co-transfection yielded no PLA signals (Fig. 5d). We also observed no significant Scube2/Gpc6 co-localization at the surface of Capan1, B16F10 and Panc1 cells, but scattered PLA signals in HeLa cells and clustered intense signals in MiaPaca2 and Bosc23 cells, consistent with their increased relative Scube2 responsiveness (Fig. 5a). We thus conclude that HS-mediated Scube2 co-localization with its substrate serves as a prerequisite for Shh release and signaling from the producing cell surface.

### Scube2 function also depends on HS binding of the substrate

The above-described mechanism predicts that both the release factor and the substrate must associate with the same HS and that the release of lipidated substrates not associated with HS is unresponsive to Scube2. To test this possibility, we expressed lipidated variants of otherwise soluble Halotag proteins in Bosc23 cells. Halotag is a modified haloalkane dehalogenase tag with a low pI of 4.89, likely preventing HS interaction of the protein. We confirmed this prediction with HS affinity chromatography by using the above-described method and column (Fig. 6a). In contrast to control Shh, soluble Halotag did not bind to HS or heparin, suggesting that lipidated Halotag will not significantly associate with cell-surface HSPGs and HSPG-associated Scube2 proteins. We expressed Halotag reporters fused to the eight most C-terminal amino acids of the N-terminal Shh signaling domain^35^ and the C-terminal Shh cholesteryltransferase domain (Halo-ShhC, Fig. 6b). We also analyzed Halo-ShhC variants lacking the ShhN peptide (Halo-ShhC^Δ190–197^) or the one lysine in this peptide (Halo-ShhC^K195G^). This peptide can be cleaved, as shown for Shh^C25A;HA^ (Fig. 1c). Still, Scube2 did not significantly increase Halotag solubilization from the Bosc23 cell surface: Scube2 did not shift the ratio between released soluble Halotag and the lipidated variants (Fig. 6b, bottom). This result leads us to suggest that the role of Hh accumulation at sites of HSPG expression^18^ is to allow for its later co-localization with Scube2 as a prerequisite for regulated Hh release from the cell surface.

## Discussion

Cellular communication requires dedicated machineries to relay information from producing to receiving cells: Regulated access of ligand to receptor is key to controlling these systems. One way to limit distant receptor interactions is to tether ligands to the plasma membrane of the cell that produces them. This tethering mechanism restricts signaling to directly adjacent cells^48^. In contrast, soluble secreted proteins signal, in a paracrine fashion, to nonadjacent cells. The more complex alternative of combined short-range and long-range signaling exploits the characteristics of both mechanisms. Consistent with their short-range signaling activities, all Hhs are dual-lipidated proteins that tether to the outer membrane leaflet^7^,^35^,^49^. However, soluble Hh molecules also exist, and N-lipidation paradoxically increases their long-range signaling activity: In transwell assays, non-palmitoylated Shh is less active than dually lipidated Shh, and non-palmitoylated fly Hh is inactive^12^,^50^. In the mouse, however, ectopic overexpression of non-palmitoylated Shh proteins induces gain-of-function phenotypes, although these are less severe than phenotypes produced by wild-type Shh overexpression^14^,^15^,^17^, and loss of palmitoylate activity causes long-range signaling defects that are characteristic of defective Shh signaling but, again, are less severe than those in Shh mutants^15^. As a result of these equivocal findings, the molecular mechanisms by which Hh spreads and signals to target cells, as well as the essential role of Hh lipids in this process, are controversial.

A first step in decoding Hh solubilization, while making the fewest assumptions, is to ask how other membrane-associated molecules, such as EGF receptor (EGFR) ligands and Wnt family members, are released from their producing cells. In flies, the EGFR ligands Spitz, Gurken, and Keren are synthesized as membrane-bound precursors, which are shed from the cell surface by integral membrane proteases called rhomboids^51^. Spitz signaling is regulated by the spatial separation of endoplasmic reticulum-resident Spitz ligands from Golgi-resident rhomboids, as well as by a trafficking partner called Star that escorts Spitz to the Golgi, where it is cleaved^52^. The basis for *Drosophila* EGFR activation is therefore to keep EGFR ligands and their sheddases apart until signaling is required. Mechanisms of release regulation are more complex in mammals, yet the logic of regulated trafficking and compartmentalization is the same: Although mammalian EGFR-ligand cleavage requires ADAM proteases instead of rhomboids, their activity is also controlled by enzyme trafficking and regulated access of enzyme to substrate. Such a mechanism also regulates the solubilization of membrane-associated *Drosophila* Wnt proteins. Wnts are secreted proteins characterized by the presence of palmitate covalently linked to conserved cysteine and serine residues^11^,^53^. Palmitate tethers the protein to the cell membrane^54^, but is also essential for Wnt signaling^53^. Membrane-associated Wnt is hydrolyzed by the extracellular Wnt-specific putative protease Tiki^55^ and the deacylase Notum^56^. Notably, Wnt deacylation is regulated by the HS chains of GPI-linked Gpcs that act as scaffolds to co-localize Notum and its substrate at the cell surface, providing another example of protein activity control by regulated co-localization.

These data suggest Gpc-mediated cell-surface assembly of Hh substrates with their sheddases or deacylases as one parsimonious mechanism for Hh release. The possibility of Hh deacylation, however, is difficult to envision for two main reasons: First, Hh-specific deacylases or sterol esterases are unknown, and second, the only extracellular deacylase Notum is specific for Wnt proteins but inactive on palmitate linked to the α-amino group of Shh/Hh^56^. We suggest that this unusual Hh N-acylation may actually serve to protect Hh from Notum, as amide-linked palmitate is resistant to any known deacylase. Otherwise, a situation comparable to that in palmitoyl-acyltransferase-deficient mutants would occur, resulting in variably impaired signaling, as described earlier. This would make reliable Hh signaling impossible, and it supports the remaining option that Hh may be released from the cell surface by proteolytic processing. Indeed, in the past, we have established the key role of shedding in releasing the active protein^26^,^35^,^40^,^43^,^57^. We also resolved the apparent paradox between proteolytic processing of lipidated terminal Shh peptides and the above-described essential role of palmitate^7^,^13^,^14^,^15^,^16^,^17^ by showing that the lipid controls the proteolytic removal of Shh N-terminal peptides during solubilization^40^,^43^, but has no direct receptor binding role as in the Wnts^53^. On the contrary, processing of palmitoylated Hh peptides relieves their block of Shh binding to the Ptc receptor, which explains the essential^7^ yet indirect^40^ role of N-palmitate for Shh bioactivity. Lack of palmitate leads to incomplete processing of inhibitory N-terminal peptides, resulting in inactive soluble proteins with their Ptc binding sites permanently blocked^40^. Results presented in this work confirm this mechanism: Hhat co-expression of tagged and untagged Shh variants unequivocally results in the loss of (tagged) N- and C-terminal lipidated peptides, which is increased by Scube2, linking Hh processing with its activation. This is in line with the finding that an N-terminally processed fraction of unpalmitoylated solubilized Shh^C25A;HA^ significantly increases reporter cell differentiation over baseline levels. In this case, only N-terminal Hh processing, but not any palmitate moiety per se (because it is absent), can be made responsible for Hh biofunction. We suggest that limited processing of non-palmitoylated proteins may also explain the above-described variable bioactivities of unpalmitoylated Shh forms *in vitro* and *in vivo*. Finally, we note that the same situation applies to the artificial monomeric protein ShhN, at least in our hands (Supplementary Fig. S1). Taken together, our findings strongly indicate coupled proteolytic processing, release, and activation^58^ of Hh morphogens in producing cells.

**Figure 7:**
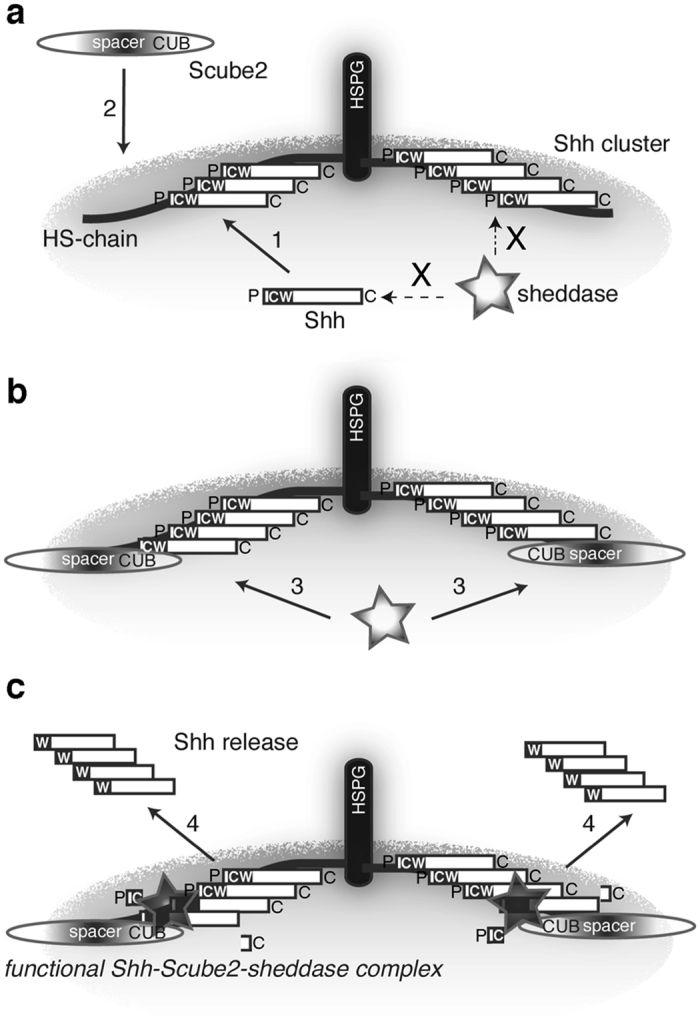
Schematic of Gpc-regulated assembly of Shh signaling hubs on the producing cell surface. (**a**) 1: Gpcs assemble Hhs into cell-surface heteroprotein hubs^18^. Activity of sheddases and sheddase activators (2) depends on their co-localization with the substrate. Otherwise, random substrate/sheddase encounters release baseline levels of substrate (indicated by an X). (**b**) Signaling is initiated by HS-dependent recruitment of soluble Scube2 to the cell surface. Shedding is then induced by the Scube2 CUB domain (3). (**c**) Functional Gpc/Shh/Scube2/sheddase signaling complexes release Shh in truncated form. The terminal lipids control this process: Continued membrane association of lipid-linked peptides ensures that only fully activated Hh clusters will leave the complex (4). P: palmitate, C: cholesterol.

However, ADAM17, the most prominent Shh processing enzyme *in vitro*^38^,^39^,^40^, processes more than 50 other substrates^37^. This raises one essential question: How can a seemingly promiscuous sheddase control Shh processing with the necessary precision? On the basis that changes in HS biosynthesis (on producing cells) affect Shh signaling^24^,^25^,^59^,^60^, we recently proposed that cell-surface Hh-associated Gpcs^18^ may be one such control protein^26^. Gpcs associate, through their GPI anchors, with lipid rafts^61^, specialized membrane microdomains that serve as local organizers for the assembly and trafficking of multiple signaling molecules and their receptors. There, Gpcs act as Hh assembly and storage scaffolds^18^, but may also recruit or activate factors required for their regulated release^26^. Our work, by identifying the soluble Hh release protein Scube2^31^,^32^ as one such HS-binding factor *in vitro*, highlights the key role of HSPGs in Shh signaling regulation by the hierarchical evolution of Shh source properties (Fig. 7). From the observation that isolated spacer and CUB domains act as dominant negative repressors of Shh processing and solubilization, whereas their physical linkage results in active “Mini-Scube2”^32^, we suggest that Scube2 bridges HSPG-associated Shh ligands with their sheddase. In this scenario, the Scube2 CUB domains may recruit or activate the Shh sheddase(s), and the spacer domain links Scube2 to HS-associated Shh. This provides a mechanistic model for Hh release from the membrane by regulated substrate/hydrolase co-localization, comparable to the situation in Wnt-producing cells and tissues^56^.

Notably, consistent with the observed specificity of HS/Scube interactions, the spacer domain shows only 46 to 50% identity between the isoforms [Supplementary Fig. S3,^62^]. This may explain Scube2 specificity for Shh^63^, despite the strong conservation of Scube1–3 CUB domains (showing 82 to 90% identity)^62^. Indeed, protease regulation by CUB domains is supported by the procollagen C proteinase enhancers PCPE1 and PCPE2 that CUB-dependently bind bone morphogenetic protein 1 (BMP1) and enhance BMP1-mediated cleavage of its substrate procollagen C^64^. Notably, PCPE1 interacts with HSPGs^65^ via a peptide next to the CUB domain, and heparin affects PCPE assembly and activation^66^. The emerging theme, therefore, is that Gpcs serve as assembly scaffolds and seeds for substrates and their enzyme linkers to establish local protease or esterase processing and substrate maturation/release hubs on the plasma membrane^26^,^56^. We suggest that decision making of these cell-surface hubs depends on substrate availability, the availability of hydrolases and adaptor proteins, and HS sulfation that regulates their assembly. In the case of the Hhs, on permissive cells such as HEK293 cells and their Bosc23 derivatives, this would result in Scube2-dependent proteolytic Hh release being favored over alternative Hh release modes. The original assumption that “Hhs are not cleaved from the cell surface, because the vast majority of Hh protein expressed in cultured cells is cell associated and not soluble”^67^ is therefore no longer valid: We note that the same statement could be made for any event (including Spitz and Wnt release) in which the relevant components for release are not expressed or otherwise available. We also propose that the more common opposite problem, that is, specific substrate release despite the presence of multiple substrates and sheddases at the surface of the same expressing cell at once, may be controlled in the same HSPG-regulated way. We will investigate this possibility.

## Methods

### Cloning and expression of recombinant proteins

Shh constructs were generated from murine cDNA (NM_009170) by PCR and ligated into pcDNA3.1 (Invitrogen) for the expression of secreted, cholesterylated 19 kDa Shh in Bosc23 cells, a HEK293 derivate. Shh^C25A^ 68, ShhN^C25A^, and GPI-linked or mycHis-tagged ShhN^69^ were generated by site-directed mutagenesis (Stratagene). Where indicated, Shh was expressed in HA-tagged form. Primer sequences can be provided upon request. Human Scube2 constructs were a kind gift of Ruey-Bing Yang^42^. Hhat cDNA (NM_018194) was obtained from ImaGenes and cloned into pIRES (ClonTech) for bicistronic Shh/Hhat and ShhN/Hhat co-expression in the same transfected cells. This resulted in N-palmitoylated, C-cholesterylated proteins or N-palmitoylated, non-cholesterylated proteins, respectively. Halotag fusion constructs with Shh were constructed as previously described^31^.

### Cell culture and protein analysis

Bosc23, B16F10, Panc1, HeLa and MiaPaca2 cells were cultured in Dulbecco’s Modified Eagle’s Medium (DMEM; Lonza) supplemented with 10% fetal calf serum (FCS) and 100 μg/ml penicillin-streptomycin and were transfected with PolyFect (Quiagen). CHO-K1 and CHO-pgsD-677 cells were cultured in DMEM/F12 (Lonza), and Capan1 cells in RPMI (Lonza). Transfected cells were cultured for 48 h, the medium was changed, and Shh was secreted into serum-free medium for 6 h or overnight. Harvested media were ultracentrifuged for 30 min at 125,000 g and proteins were TCA precipitated. Where indicated, 600 μg/ml Mβcd (Sigma) was added to serum-free media for 2 h to induce shedding. For co-immunoprecipitation, centrifuged supernatants were incubated with 5E1-coupled PA agarose beads overnight. Heparin pulldown was conducted by using 30 μl/ml heparin sepharose (Sigma). Where indicated, 300 μl of heparin-sepharose beads were preincubated for 6 h with 15 ml of poly-L-lysine (0.1% solution, Sigma) or recombinant Scube2 spacer proteins to impair full-length Scube2/heparin interactions. All proteins were analyzed by 15% SDS-PAGE, followed by Western blotting using polyvinylidene difluoride membranes. Blotted proteins were detected by α-HA antibodies (mouse IgG; Sigma), polyclonal α-FLAG antibodies (rabbit IgG; Sigma), α-Shh antibodies (goat IgG; R&D Systems), or polyclonal α-CW antibodies directed against the heparan sulfate-binding CW sequence (rabbit IgG; Cell Signaling). Incubation with peroxidase-conjugated donkey-α-goat/rabbit/mouse IgG (Dianova) was followed by chemiluminescent detection (Pierce). Signals were quantified by using ImageJ. Photoshop was used to convert grayscale blots into merged RGB pictures for improved visualization and quantification of N- and C-terminal peptide processing.

### Shh reporter assays

C3H10 T1/2 reporter cells^44^ were grown in DMEM containing 10% FCS and 100 μg/ml penicillin-streptomycin. At 24 h after seeding, serum-free Shh-conditioned media were diluted 1:1 with DMEM containing 20% FCS and 100 μg/ml antibiotics and applied to C3H10 T1/2 cells. Cells were lysed 5 to 6 days after induction (20 mM Hepes, 150 mM NaCl, 0.5% TritonX-100, pH 7.4) and osteoblast-specific alkaline phosphatase activity was measured at 405 nm after addition of 120 mM p-nitrophenolphosphate (Sigma) in 0.1 M glycine buffer (pH 9.5).

### Chromatography

HS columns were generated as follows: 1 g (wet weight) C57/B16 mouse embryos (E12-E18) were homogenized and digested in 320 mM NaCl, 100 mM sodium acetate (pH 5.5) containing 1 mg/ml pronase, and 1 mg/ml proteinase K for 72 h at 40 °C. Fresh enzymes were added every 12 h. The digested samples were centrifuged, filtered, diluted 1:3 in water and 2.5 ml aliquots were applied to DEAE Sephacel columns. Eluted glycosaminoglycans were lyophilized, diluted, and quantified by the carbazole reaction. The material was then coupled to NHS-activated sepharose columns. The columns were tested using recombinant HS-binding proteins fibroblast growth factor 2 and 8, vascular endothelial growth factor and Semaphorin 3F as positive controls. FPLC was conducted on an Äkta protein purifier (GE Healthcare). Samples were applied to the columns in the absence of salt, and bound material was eluted by using a linear 0 to 1.5 M NaCl gradient in 0.1 M phosphate buffer (pH 7.0). Eluted fractions were TCA precipitated and analyzed by SDS-PAGE as described above. Signals were quantified by using ImageJ. Gel filtration analysis was performed by using a Superdex200 10/300 GL column (Pharmacia) equilibrated with PBS at 4 °C.

### FACS

Scube2-transfected Bosc23 cells and CHO-K1 and CHO-pgsD677 cells were non-enzymatically removed from the culture dish by using Versene (PAA) and suspended in PBS containing 5% FCS in a total volume of 0.5 ml. Cells were incubated with heparinases I to III (AMS Biotechnology) at 37 °C or with 10 μg/ml heparin (AppliChem) at 4 °C for 1 h. Cells were washed and treated with α-FLAG antibody (1:500 dilution) for 1 h and fluorescein isothiocyanate-conjugated goat-α-rabbit secondary antibody (1:200 dilution, Dianova) for 30 min on ice. FACS analysis was performed on a BD Accuri C6 flow cytometer (BD Biosciences). Histograms were created by using FlowJo single cell analysis software.

### *In situ* PLA

Transfected cell lines were fixed in 4% PFA under non-permeabilizing conditions and subjected to Duolink *in situ* fluorescence detection (Sigma) according to the manufacturer’s instructions. Briefly, slides were blocked; incubated with primary antibodies directed against tagged Gpc6 (α-HA antibodies, mouse IgG; Sigma), tagged Scube2 (α-FLAG antibodies, rabbit IgG; Sigma) and Shh (α-Shh antibodies, goat IgG; R&D Systems); washed; and incubated with secondary antibodies conjugated to oligonucleotides (PLA probes, Sigma). Circularization and ligation of the oligonucleotides was followed by an amplification step with nucleotides and fluorescent oligonucleotides. Negative controls always included transfected cell lines expressing both target proteins. These cells were incubated with each single primary antibody and both PLA probes. Slides were mounted with Duolink *in situ* mounting medium and evaluated by using an LSM 700 confocal microscope (Carl Zeiss). Z-stack micrographs taken with 40/63 objectives were obtained. Representative results are shown from experiments repeated at least twice.

### RT-PCR

For RT-PCR analysis of dispatched mRNA expression, TRIzol reagent (Invitrogen) was used for RNA extraction from cultured cells, and a first-strand DNA synthesis kit (Thermo, Schwerte, Germany) was used for cDNA synthesis. PCR was performed for 35 cycles by using intron-spanning primer pairs (sequences can be provided upon request).

### Bioanalytical and statistical analysis

Sequence analysis was conducted on the CFSSP secondary structure prediction server (http://www.biogem.org/tool/chou-fasman/). All statistical analysis was performed in GraphPad Prism by using the Student’s *t* test (two-tailed, unpaired, confidence interval 95%). All error estimates are standard deviations of the mean (s.d.).

## Additional Information

**How to cite this article**: Jakobs, P. *et al*. Bridging the gap: heparan sulfate and Scube2 assemble Sonic hedgehog release complexes at the surface of producing cells. *Sci. Rep.* **6**, 26435; doi: 10.1038/srep26435 (2016).

## Supplementary Material

Supplementary Video 1

Supplementary Video 2

Supplementary Video 3

Supplementary Video 4

Supplementary Information
