# Supplementary material for: Bridging the gap: heparan sulfate and Scube2 assemble Sonic hedgehog release complexes at the surface of producing cells
Source: Sci Rep. 2016 May 20;6:26435. doi: 10.1038/srep26435 (PMC4873810; doi:10.1038/srep26435)
Supplement: Supplementary Information [file srep26435-s5.pdf]

Supplementary Materials for

Bridging the gap: heparan sulfate and Scube2 assemble Sonic Hedgehog release complexes at the surface of producing cells

P. Jakobs[1], P. Schulz[1], C. Ortmann[1], S. Schürmann[1], S. Exner[1], R. Rebollido-Rios[2], R. Dreier[1], D. G. Seidler[1], K. Grobe[1*]

Corresponding author: kgrobe@uni-muenster.de

This file contains:

Fig. S1: Scube2 activates shedding of membrane-anchored, cholesterol-unmodified ShhN.

Fig. S2: HS and heparin binding of Scube1-3.

Fig. S3: Protein sequence alignment of the Scube1-3 spacer region.

Fig. S4: FACS analysis reveals different retentions of Scube2 depending on cell-surface HS.

Fig. S5: CHO-K1 cells are Scube2 insensitive, yet secrete Scube2 and express Shh substrates and sheddases at the cell surface.

Movies:

Confocal images of cell-surface PLA signals were taken on a Zeiss LSM700 microscope using a 63× objective. PLA M1 and PLA M2 show Shh/Scube2 interactions at the cell surface; PLA M3 and PLA M4 show interactions between Gpc6 and Mini-Scube2.

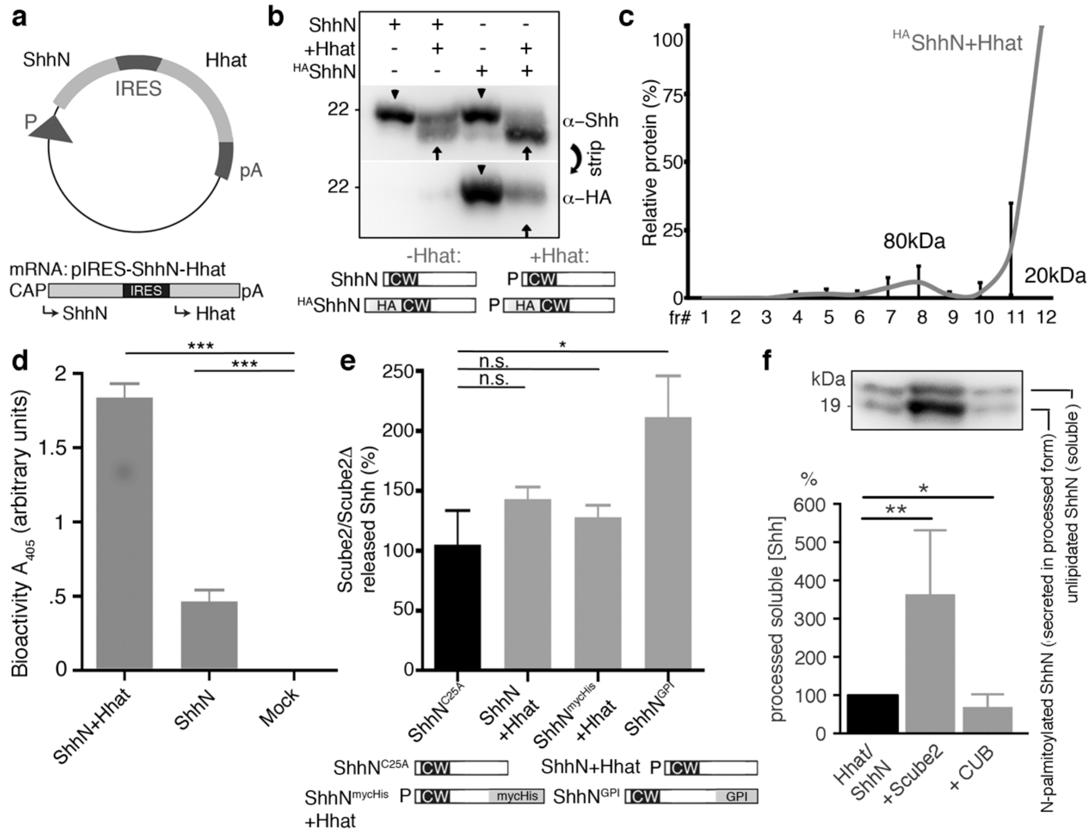

Fig. S1: Scube2 activates shedding of membrane-anchored, cholesterol-unmodified ShhN. a) pIRES-coupled ShhN/Hhat co-expression was employed to generate N-palmitoylated, uncholesterylated proteins. Shh translation from bicistronic mRNA was CAP dependent, whereas Hhat translation was CAP independent. P: promotor, IRES: internal ribosomal entry site, pA: polyadenylation signal. **b)** ShhN and N-terminally HA-tagged ^{HA}ShhN were expressed in Bosc23 cells in the presence or absence of Hhat and the soluble fractions analyzed by immunoblotting. Hhat co-expression resulted in ShhN processing during release (arrows), as demonstrated by a molecular weight shift and loss of α–HA antibody reactivity of the tagged form. Lack of exogenous Hhat expression led to the secretion of largely unprocessed proteins (arrowhead). Bottom: Schematic of ShhN/ ^{HA}ShhN posttranslational lipidations in the presence or absence of Hhat. **c)** Gel filtration analysis of ShhN expressed in the

presence of Hhat. The solubilized protein is largely monomeric, an observation incompatible with continued lipidation of soluble ShhN. We interpret the small fraction of detectable oligomeric 80kDa proteins as a consequence of lipid-independent, yet HS-assisted morphogen clustering at the surface of the producing cells. **d)** Hhat co-expressed ShhN and ShhN expressed in its absence differentially induced Hh-dependent C3H10T1/2 reporter cell differentiation into alkaline phosphatase producing osteoblasts. ShhN expressed in the absence of Hhat showed strongly reduced biofunction (Shh+Hhat: 1.84±0.1 arbitrary units (au), n=6, p≤0.0001, ShhN: 0.47±0.08 au, n=6, p≤0.0001 if normalized to mock values). **e)** If compared to unlipidated $ShhN^{C25S}$ control proteins directly secreted from the cell surface (black bar), Scube2 enhanced the release of N-palmitoylated ShhN (136% and 122% in two independent experiments) as well as the release of a ShhN construct C-terminally fused to the human CD55 GPI target sequence (+202%). **f)** Full-length Scube2 increases ShhN release from producing cells, but the CUB domain represses release below base-line amounts set to 100%. Scube2 364%±68%, p=0.0031 **, n=6; CUB 69%±13%, p=0.045 *, n=6.

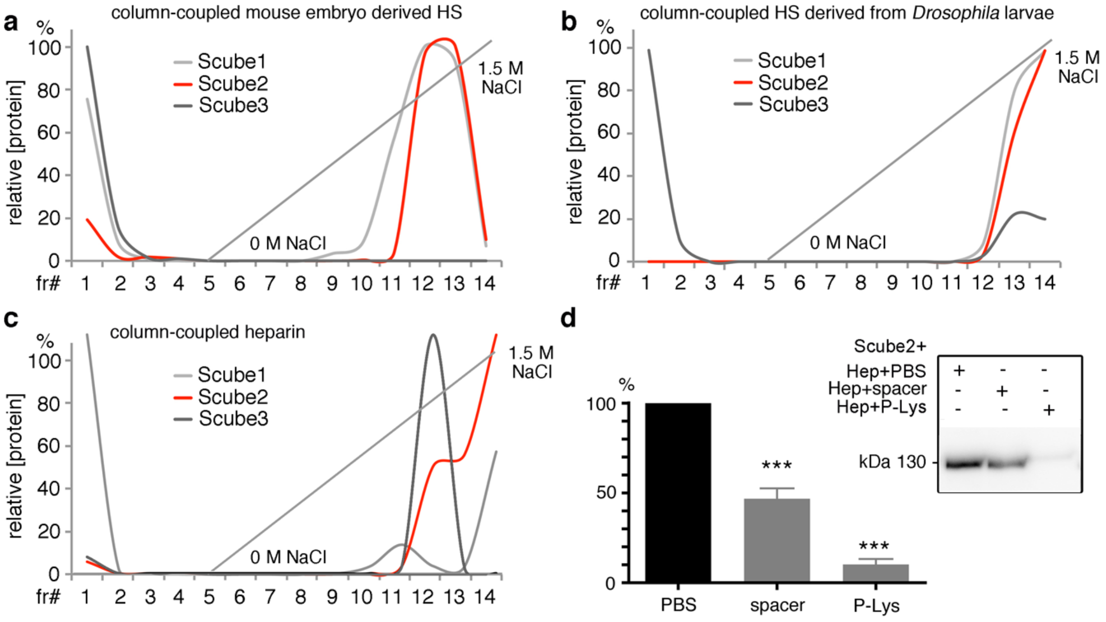

Fig. S2: Scube1 and Scube2, but not Scube3, bind strongly to HS. a,b) Scube1 and Scube2 family members strongly bind to mouse embryo-derived HS and to HS derived from *Drosophila melanogaster* larvae. In contrast, Scube3 binding to both columns was strongly impaired. This observation is in line with robust Scube1 and Scube2 activities, but only moderate Scube3 activity, in Shh release from Shh-LIGHT2 cells, a mouse embryo-derived cell line, and from HEK293T cells [1]. Note that Scube3 interacted weakly with invertebrate HS, but strongly with heparin **(c)**. Mouse embryo-derived HS consists of multiple unmodified N-acetylated domains, highly N-sulfated domains, and mixed domains consisting of both domain types [2]. *Drosophila* HS consists of a single extended N-sulfated domain [3], and heparin represents a highly and continuously sulfated form of HS. Variable binding to these differently sulfated glycosaminoglycans thus indicates that Scube3/HS interactions specifically require a high degree of continuous HS sulfation. **d)** Equal amounts of soluble Scube2 were subjected to heparin-sepharose pulldowns, and the precipitates were subjected to SDS-PAGE and immunoblotting. Heparin-sepharose preincubation with Scube2 spacer domains (spacer) or poly-lysine (P-Lys) reduced Scube2 binding

to heparin, demonstrating the specificity of the interaction. The amount of Scube2 pulled down by heparin-sepharose in the absence of any competing proteins was set to 100%. Scube2 pulled down by spacer-preincubated heparin-sepharose: 47%±6%; Scube2 pulled down by P-Lys-preincubated heparin-sepharose: 10%±3%, ***p=0.0001, n=5.

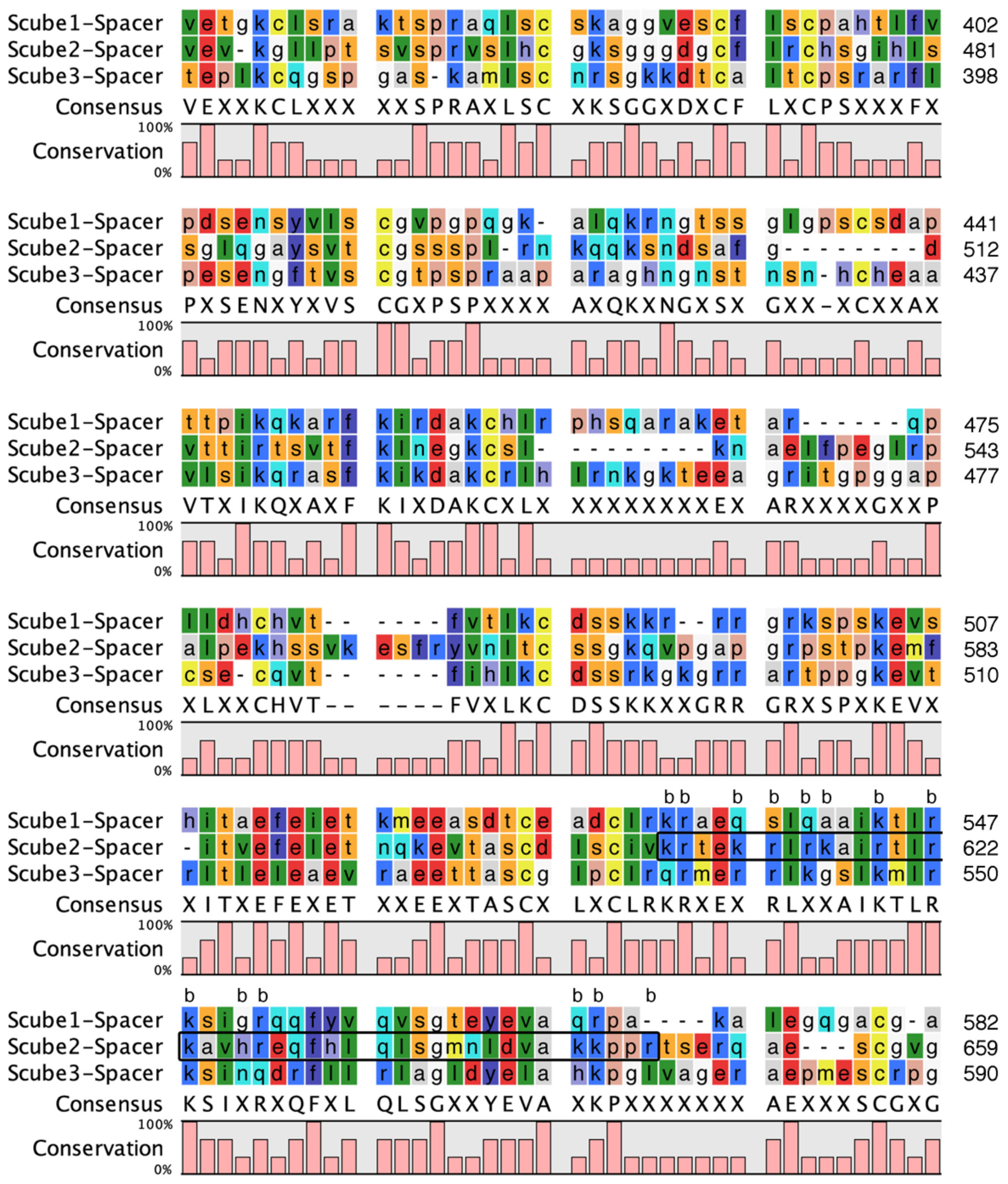

Fig. S3: Protein sequence alignment of the Scube1-3 spacer region. The HS-binding amino acid cluster mutated in Spacer-/Scube2ΔHS1 and Scube2ΔHS2 is boxed.

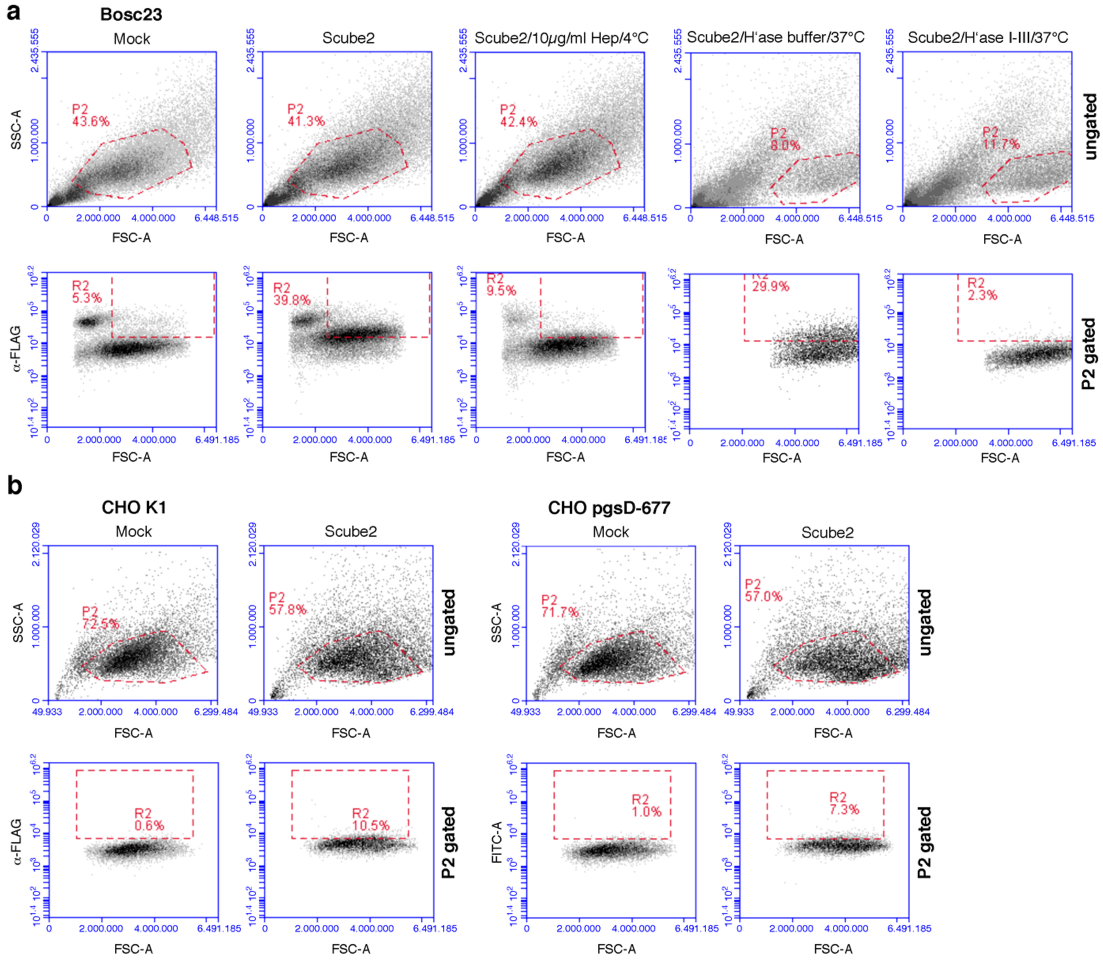

Fig. S4: FACS analysis reveals different retentions of Scube2 depending on cell-surface HS. a) Bosc23 cells. b) CHO-cells. FACS plots of Scube2-expressing and control Bosc23, CHO K1 and CHO pgsD-677 cells. Top: 50,000 cells were analyzed per assay (representing the gated P2 fraction). Bottom: The P2 fraction was analyzed for relative amounts of Flag-tagged Scube2 at the cell surface under various experimental conditions. The R1 fraction was subtracted from 7AAD-stained dead cells (not shown).

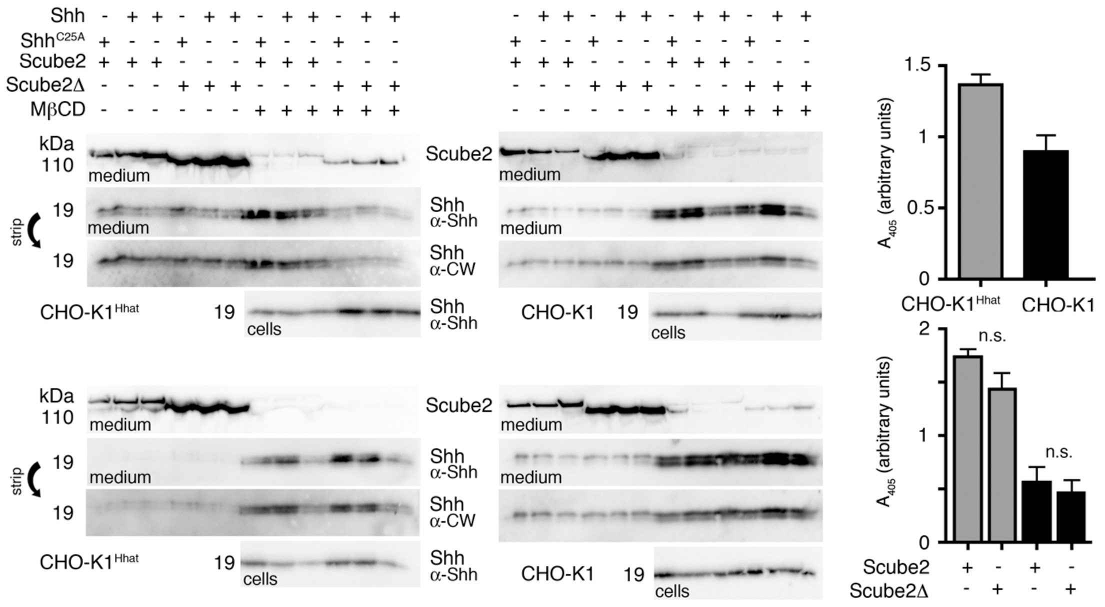

Fig. S5: CHO-K1 cells are Scube2 insensitive, yet secrete Scube2 and express Shh substrates and sheddases at the cell surface. Scube2 or Scube2Δ was expressed in CHO-K1 cells stably transfected with Hhat (left) or CHO-K1 wild-type cells that also expressed Shh or Shh^{C25A}. 36h post transfection, cells were washed and incubated for 6h under serum-free conditions. After the medium was harvested, we added 600μg/ml MβCD to the same cells to release the previously unreleased surface-bound material. This step also confirmed the presence of Shh and Shh sheddases at the cell surface. In the absence of MβCD, Scube2 released only little Shh and Shh^{C25A} amounts from the CHO-K1 cell surface into serum-free media. Morphogen release was strongly enhanced by MβCD, and Shh released from MβCD-treated CHO-K1 wild-type and Hhat-transfected cells was bioactive (right top). No significant effect of Scube2 co-expression on the release of bioactive Shh in the presence of MβCD could be observed (bottom right).

References

1 Lin, Y.-C., Roffler, S. R., Yan, Y.-T. & Yang, R.-B. Disruption of Scube2 Impairs Endochondral Bone Formation. *Journal of Bone and Mineral Research* **30**, 1255-1267 (2015).

2 Carlsson, P., Presto, J., Spillmann, D., Lindahl, U. & Kjellen, L. Heparin/heparan sulfate biosynthesis: processive formation of N-sulfated domains. *J Biol Chem* **283**, 20008-20014 (2008).

3 Kusche-Gullberg, M., Nybakken, K., Perrimon, N. & Lindahl, U. Drosophila heparan sulfate, a novel design. *J Biol Chem* **287**, 21950-21956 (2012).
